# Relationship Between the Self-Concept of Children and Their Ability to Recognize Emotions in Others

**DOI:** 10.3389/fpsyg.2021.672919

**Published:** 2021-10-12

**Authors:** Teresa Cordeiro, Júlia Botelho, Catarina Mendonça

**Affiliations:** ^1^Department of Psychology, Faculty of Human and Social Sciences, Azores University, Ponta Delgada, Portugal; ^2^Center for Psychology, Faculty of Psychology and Education Sciences, Porto University, Porto, Portugal

**Keywords:** social cognition, facial expressions, emotion recognition ability, academic self concept, PERT, Piers-Harris

## Abstract

The aim of this study was to assess the relationship between the self-concept of children and their ability to recognize emotions in others from facial expressions. It is hypothesized that children use their self-representations to interpret depictions of emotion in others and that higher self-concepts might be associated with earlier development of emotion recognition skills. A total of 54 children aged between 5 and 11 years participated in this study. Self-concept was assessed in all children using the Piers-Harris Self-Concept Scale for Children (Piers-Harris 2). To assess emotion recognition, a computerized instrument, the Penn Emotion Recognition Task (PERT), was applied. Despite the small sample of children, results show clear statistical effects. It is shown that emotion recognition ability is directly correlated with self-concept for intellectual/school status. The ability to correctly identify emotions from facial expressions is affected by general self-concept, intellectual/school status, and stimulus features of gender, intensity, and emotion. Further analysis shows that the general self-concept of children particularly affects the ability to identify happy faces. Children with a higher intellectual status score recognize happiness and neutral faces more easily. We concluded that the self-concept in children relates to the ability to recognize emotions in others, particularly positive emotions. These findings provide some support to the simulation theory of social cognition, where children use their own self-representations to interpret mental states in others. The effect of the self-concept for intellectual status on emotion recognition might also indicate that intellectual abilities act as a mediator between self-concept and emotion recognition, but further studies are needed.

## Introduction

Social cognition is a neurobiological, psychological, and social set of processes. It is thought that this set of skills affects how social events are perceived, recognized, and evaluated in order to build representations that can guide social behavior (Adolphs, 2010). Understanding emotions from facial expressions is a specific form of social cognition. Facial expressions are a prestigious form of nonverbal social communication. The information extracted from a facial expression is crucial for the proper functioning of interactions and interpersonal relationships (Hinojosa et al., 2015), possibly because facial expressions are one of the main forms of emotion manifestation (Ekman, 2011).

According to Lewis et al. (2008), adults tend to organize emotions in a hierarchy where the biggest categories (which are on a superordinated level) are subdivided into more specific, basic-level groups. Those basic groups are once again divided into even more specific groups (presented on a subordinated level). They also mentioned that it is likely that children start by recognizing emotions on a basic level such as “Happiness,” “Surprise,” “Fear,” “Anger,” “Disgust,” and “Sadness.” Later, they started to comprehend that those emotions can be subdivided into a subordinate level (e.g., “Surprise” is subdivided into “Startle” or “Shock”).

Emotion recognition through facial stimuli has been robustly demonstrated in the literature (Adolphs, 2002b; Gur et al., 2002; Ruiz-Ruiz et al., 2006; Aviezer et al., 2008; Zaja and Rojahn, 2008; Pollak et al., 2009; Harms et al., 2010; Segal et al., 2019). The recognition of facial emotions is understood as a process in which facial characteristics are perceived and analyzed as a means of identifying a specific emotional state (Adolphs, 2002a,b). The recognition of facial expressions does not appear at a specific stage of development. This is a gradual process, where this ability emerges over time. However, happiness is recognized more precisely beforehand, followed by sadness and anger. Regarding the expressions of surprise, fear, and disgust, these end up being better recognized at later moments (Lawrence et al., 2015). The capability of recognizing what a person is feeling by their facial expression might be affected by the intellectual abilities of individuals (Zaja and Rojahn, 2008) and other mental disorders (Harms et al., 2010, Kohler et al., 2003). According to a study by Nelson and Mondloch (2019), which compared emotion perception in children and adults, adults are more accurate than children, but the pattern of visual attention during the task does not differ. Segal et al. (2019) studied emotion recognition from facial expressions of individuals of different races. They determined that children are less precise in identifying emotions than adults. Participants had more success in identifying happy facial expressions, and it was possible to identify a correlation between age and emotion recognition. The intensity of emotion expression is another important factor that may influence the recognition of a certain facial expression (Rosenberg et al., 2015). Expressions associated with emotions of anger and disgust have strong similarities in terms of their facial configuration. As such, Aviezer et al. (2008) highlighted the body influence for the recognition of emotions with a high degree of similarity.

Vosk et al. (1983) compared children who were either considered socially accepted or socially rejected, according to a classification based on peer metrics and peer sociometrics. They concluded that children considered socially accepted had a higher rate of success in emotion recognition than their peers. This can be a result of social interaction problems that affect diverse neurological development areas of children such as cognition, perception, and behavior. There is a body of research that also shows how early experiences can shape the ability to identify emotions in others. In particular, abused children do better at identifying facial expressions of anger (Pollak and Sinha, 2002; Pollak et al., 2009) but do worse at identifying other emotions and positive emotions in particular (Koizumi and Takagishi, 2014). These findings raise the question about whether children with different levels of ability to recognize emotion in others will also have lived different levels of positive or negative experiences and, therefore, have a different self-image or self-concept.

Insights from neuroscience seem to support the hypothesis that the construction of the self and the perception of others are two mental processes that share the same basis, similar to two sides of the same coin (Uddin et al., 2007). Regarding motor behavior, the mirror-neuron system provides the connection between self and others, through motor simulation (Rizzolatti and Craighero, 2004; Iacoboni, 2005). Concerning the subjective construction of the self, the cortical midline structures are involved in processing both information about the self and others, in evaluative terms. These structures involve the medial prefrontal cortex, the anterior cingulate cortex, and the precuneus and have been associated with both self-processing (Northoff and Bermpohl, 2004) and social cognition (Schilbach et al., 2006). More specifically, the cortical midline structures are engaged while performing tasks, such as self-referential judgments, self-appraisal, and judgments of own personality traits (Gusnard et al., 2001; Kelley et al., 2002; Lou et al., 2004; Ochsner et al., 2005), and they are also activated during watching social interactions, observing facial expressions, forming social judgments, or tasks that require identifying social traits of others (Gobbini et al., 2004; Iacoboni et al., 2004; Mitchell et al., 2005; Uddin et al., 2007).

This functional overlap may signify that humans use their own experiences to infer the mental states of others. This hypothesis has been formalized before, as the simulation theory. Proponents of this theory (first proposed by Gordon, 1986; Adolphs, 2002a; Mitchell et al., 2005; Shanton and Goldman, 2010) argue that one of the main strategies for inferring the mental states of others would be to imagine own thoughts, feelings, or behaviors of individuals in a similar situation as theirs. This mechanism would result from the fact that, while we have no access to the minds of other people, we do enjoy direct, continuous access to our own mind. Within this theory, it is conceivable that self-representations might affect how we perceive others, at various levels. The way we see ourselves, and which emotions we experience, would therefore affect what we assume as the mental processes and emotions of others.

Self-concept involves a deep construction of the self. This construction, which can also be conceptualized as a self-development process, has been analyzed by different areas of knowledge, such as psychology, sociology, and education. Different approaches agree that there is a progressive construction of the self during childhood, which is influenced by established interpersonal relationships and by the surrounding environment (Carneiro et al., 2003; Castro et al., 2015; Harter, 2015; Muniz and Fernandes, 2016; de Oliveira et al., 2017). Family exerts a strong influence on self-evaluations constructed by children. It is from the family environment that children build their primary self-concepts, which constitute a foundation for secondary self-concepts, which in turn are created through relationships beyond the family (Resett et al., 2016). According to a study by Dunn and Cutting (1999), recognition of the emotions of 4-year-olds was positively correlated with the quality of their interactions with close friends (such as communication or good cooperation). In another study by Edwards et al. (1984), the accuracy in identifying facial expressions of emotions was correlated with popularity. Cassidy et al. (1992) in their study concluded that the emotional understanding of children and their popularity with peers during the first year of obligatory schooling were positively correlated. All these results indicate that good emotion recognition is a good indicator of the quality of their social relationships. Beyond the influence of social interactions, low levels of self-concept can also be associated with a low level of academic development, behavioral concerns, and mental health concerns (Cheong et al., 2016).

The self-concept of children involves structures that are a fundamental aspect of their mental health, such as a feeling of high personal value, which is associated with greater psychological well-being (Harter, 2012; Resett et al., 2016). Self-concept is related to the judgment of individuals of how positive or negative all areas of life are. Therefore, it may project all the expectations of individuals as a way of interpreting the lived experiences (Harter and Leahy, 2001). This construct encompasses great multidimensionality, and insofar, it is possible to identify the following four dimensions where the child tends to evaluate the self: social self-concept, scholar, familiar, and personal (Carneiro et al., 2003; Muniz and Fernandes, 2016). Furthermore, this concept is not static, as it can be changed over time due to lived experiences and to how significant the individual considers them (Harter, 1996; de Oliveira et al., 2017). According to Harter and Leahy (2001); Harter (2015), children aged between 8 and 11 years are capable of self-description when it comes to different areas of their lives, which reveals that they already have created a model of themselves, not only based on their own representations and evaluations but also by the evaluations of others (Resett et al., 2016). Children of this age also differentiate the various domains of self-concept (Jacobs et al., 2002).

In this study, we aimed to examine the association between self-evaluations of children and their ability to recognize emotions in others, specifically concerning the population of primary school-aged children, since there was a lack of studies in this field. This study aimed to analyze how the aspects of self-concept might affect social cognition in primary school-age children, particularly their ability to discriminate emotions from facial expressions. Could the way children see themselves relate to the way they perceive depictions of the emotions of others? Could certain aspects of their self-concept relate to emotion perception ability, but not others? It was hypothesized that children use their self-representations to interpret depictions of emotion in others and that higher self-concepts might be associated with earlier development of emotion recognition skills. To address these questions, a study was devised where children were assessed both in an emotion recognition task and in their self-concept.

## Materials and Methods

### Participants

The study was conducted with the participation of children from two educational institutions (*n* = 54, group 1 *n* = 20, group 2 *n* = 34). Ages of participants ranged between 5 and 11 years (mean = 8.31; δ = 1.941), of whom 25 (46.3%) were males and 29 (53.7%) were females. The participants who were 5 years old were few and turned 6 in the year in which the data collection was carried out. All participants were already attending primary school and were able to read. All participants were ethnically Azoreans.

All those legally responsible for the participants were asked to authorize their participation in this study through free and informed consent, where their voluntary participation was assured, as well as the anonymity and confidentiality of the data were obtained. All the procedures used to execute this investigation were approved by the Ethics Committee of the University of the Azores. Authorizations, as well as free and informed consents were sent to the teachers of each class so that they could send them to the parents or legal representatives of the children. Only children with the authorization signed by their primary caretaker were included in this study. During the application of the instruments, the teacher of each class brought the children to the room where the data were collected, and at the end, the teacher took them back to their colleagues in another space of the school.

### Materials

Two instruments were used to achieve the goals of this study. To assess self-concept, the Piers-Harris Self-Concept Scale for Children (Piers-Harris 2) (Piers and Herzberg, 2002) was used to measure the self-concept through 60 items, divided into 6 factors as follows: behavioral aspect, intellectual and school status, appearance and physical attributes, anxiety, popularity, and satisfaction and happiness. The Piers-Harris 2 total score ranges from 0 to 60 points. For a given item, the score varies between 1 and 0 points. This is assigned according to the answer given, where 1 corresponds to a positive attitude toward self and 0 to a negative attitude. The general self-concept is the result of the sum of the six factors of the scale. In this study, the Cronbach's alpha was 0.78, although, in the original study, it had a value of 0.90.

To assess emotion recognition of children, we used the Penn Emotion Recognition Task (PERT), an instrument based on the work developed by Gur et al. (2002) based on the three-dimensional facial expressions computerized to make children correspond the facial expression to one of the following emotions: happy, sad, fear, surprised, or no emotion. A total of 40 different facial expressions were presented for each participant, corresponding to the 5 emotions, 2 genders, 2 intensities (i.e., low and high), and 2 examples per case. The children were initially subjected to a familiarization trial. In each trial, there were five response options that correspond to the five emotions mentioned, and the child would have to select the emotion that best described the facial expression, using a mouse interface. After the familiarization trial, they would then have 40 trials, each with one facial expression. In the end, the children had access to the number of correct answers that could vary from 0 to 40. It should be noted that there was no time limit to complete the task. The stimuli were presented in random order. There was no specific order of application of the two instruments.

### Procedure

The data were collected between July and November 2019 in person at two institutions. The self-report questionnaire (Piers-Harris 2) was administered to the participants in the paper form and took 7–10 min for the older participants and ~20 min for the younger participants. In the case of very young children, the researchers read and instructed the instruments so that they were easy to understand. The PERT was administered in the digital format through the use of a computer with a 15-inch screen, and its application took no longer than 15 min per child. The aim of this study was to evaluate the children of all ages attending primary school education, although the instrument is only validated for people aged 7 years (2nd year of schooling) and older. We decided to use this instrument so that the same instrument would be used for the entire sample. It should be noted that the sample included few children who had not yet turned 6 years at the time of data collection, and all children were able to read.

The data were analyzed through the statistical software IBM Statistical Package for the Social Sciences (SPSS) version 24 and Microsoft Excel (IBM Corp, 2017; Microsoft Corporation, 2018). A descriptive analysis of the data was made, using means and SDs. Differential tests were used to compare girls and boys, namely the independent samples *t*-test for the normally distributed variable and the Mann–Whitney *U* test for the non-parametric, non-normally distributed variable. Correlation tests of Pearson (for the normally distributed variables) and Spearman (for the non-normally distributed variables) were conducted, in order to understand possible associations between the two constructs that constitute this study: self-concept scores and emotion recognition total scores. To compare the results according to the emotions presented in the stimuli, a one-way ANOVA was applied, to the correct recognition score per participant per emotion. It would not be possible to use a repeated-measures ANOVA, since there were only two repetitions per stimuli, and data per trial were binomial in nature (correct/incorrect). To analyze in greater detail the factors contributing to emotion recognition and due to the categorical nature of the data (correct vs. incorrect recognition), binomial logistic regressions were performed. To verify the normality of the data, we applied the Kolmogorov–Smirnov test. It was observed that self-concept data were not normally distributed, while emotion recognition scores were. As a way to avoid missing values, we always ensured that all answers were given.

## Results

### Age and Gender Analyses

First, we compared girls and boys regarding their performance in the two assessment measures. It was observed that girls did not differ from boys in their ability to recognize emotions [*t*_(52)_ = −0.834, *p* = 0.957, *d* = 0.23], although girls had a slightly higher average score in the task (proportion of correct answers = 0.726) than boys (proportion of correct answers = 0.701). Regarding self-concept, it was observed that girls had a slightly higher overall self-concept (49.17) than boys (46.92). However, there were no significant differences in self-concept between the two groups (*U* = 411.5, *p* = 0.395, *r* = 0.117). Therefore, all data were analyzed together.

**Figure 1:**
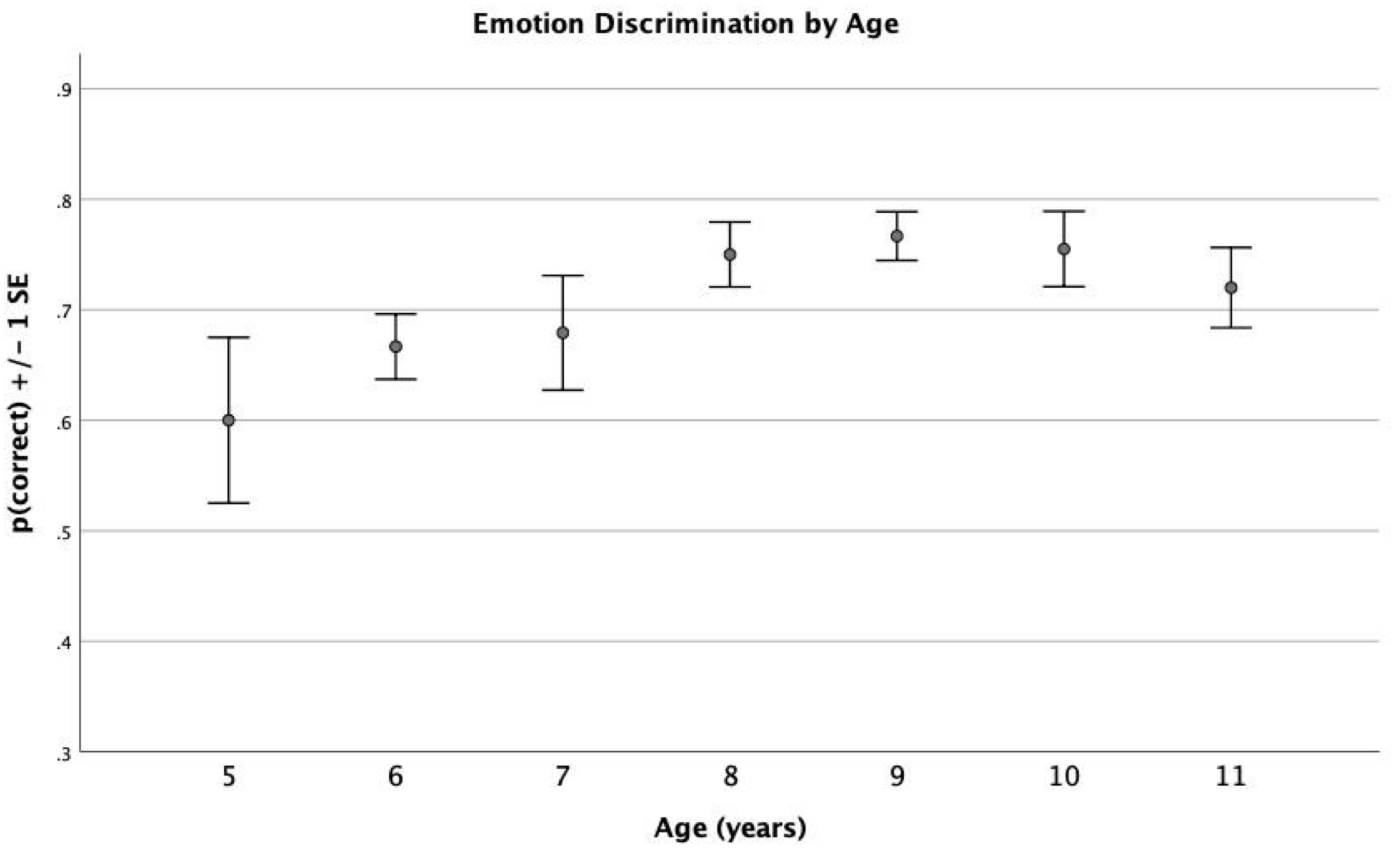
Proportion of correct emotion detections by the age of children.

It was found that emotion recognition ability increased with age (Figure 1), in particular, aged between 5 and 8 years. There was a weak positive correlation with the Pearson test between age and emotion recognition (*r*^2^ = 0.284, *p* = 0.037). This means that, as the age increases, the other variable also increases. In fact, the emotion discrimination scores gradually increase from 0.6 at the age of 5 years to 0.75 at the age of 8 years, peaking at 0.76 at the age of 9 years. Regarding self-concept, it was found that there was a significant moderate negative correlation with age (*r*_*s*_ = −0.391, *p* = 0.003). Therefore, younger participants revealed a higher self-concept than older participants. Between 5 and 8 years of age, participants had an above-average self-concept (scores of 51, 51, 52, and 54 for the ages of 5, 6, 7, and 8 years, respectively), and between 9 and 11 years of age, they had a self-concept below average (scores of 40, 43, and 42 for the ages of 9, 10, and 11 years, respectively).

### Self-Concept and Emotion Recognition Analyses

**Figure 2:**
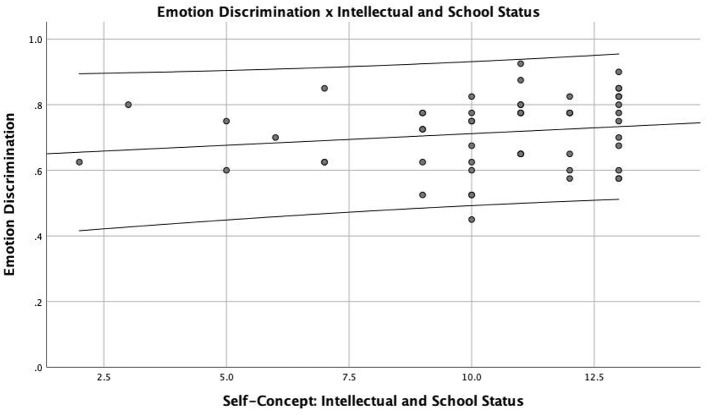
Association between correct emotion discrimination and self-concept for intellectual and school status.

Regarding the relationship between self-concept and emotion recognition, there was a weak positive correlation between the variables, namely, the intellectual/school status and general emotion recognition scores (*r*_*s*_ = 0.233; *p* = 0.045) (Figure 2). There were no statistically significant correlations between general emotion recognition and the remaining factors of self-concept (i.e., behavioral aspect, appearance and physical attributes, anxiety, popularity, and satisfaction and happiness), neither for the total self-concept score (*r*_*s*_ = 0.043; *p* = 0.378).

**Figure 3:**
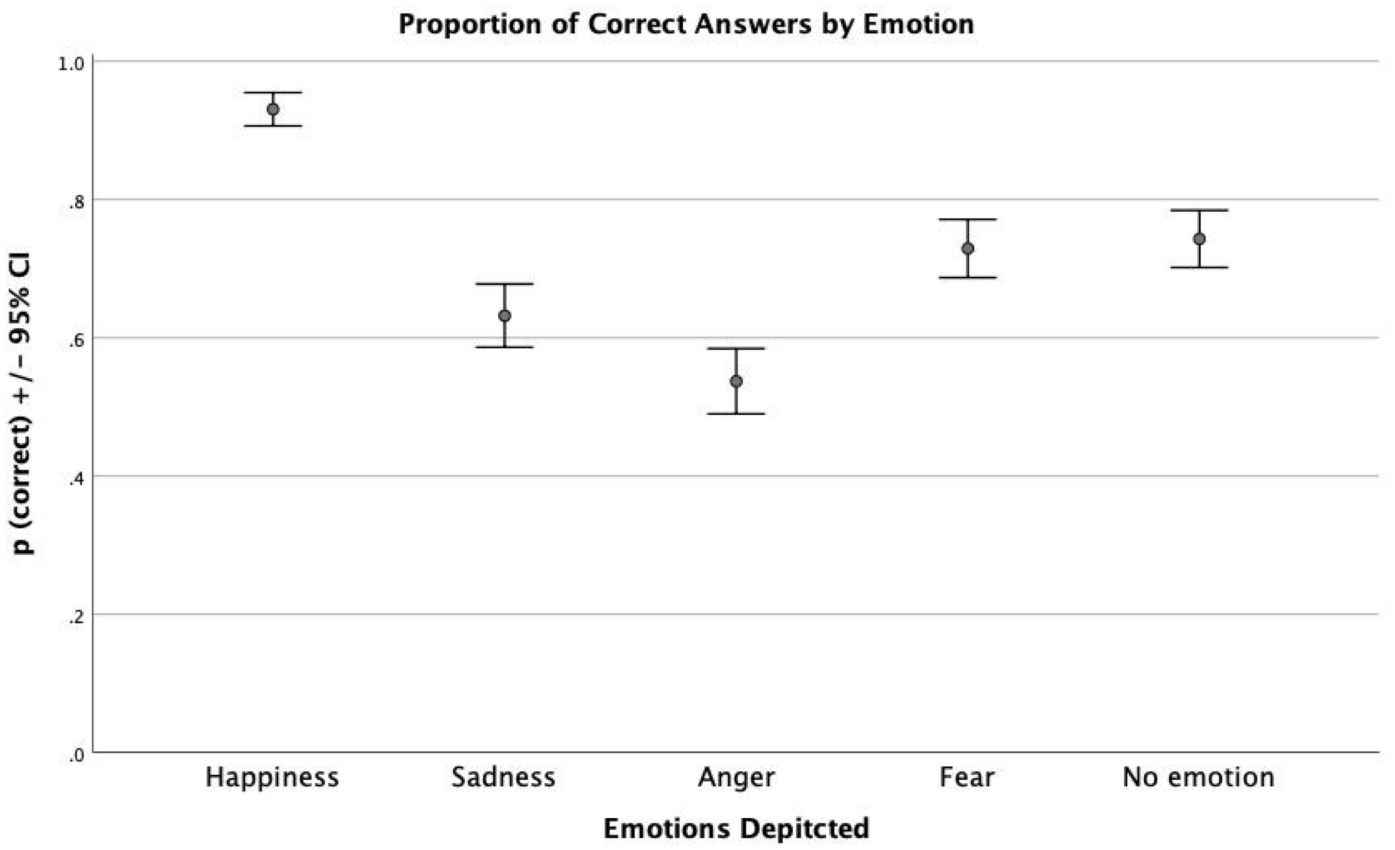
Proportion of correct emotion discriminations by emotion depicted.

To understand how the recognition of emotions varies according to the emotion in question, a one-way ANOVA was applied. There was a medium-size effect of emotion on the success rate [*F*_(4)_ = 49.604, *p* = 0.000, η^2^ = 0.84]. As shown in Figure 3, happiness was the emotion most easily identified by the children, with a 93% success rate. Anger was the emotion with the lowest percentage of correct answers (54%). However, fear and no emotion were equally as easily identified by children. According to the *post-hoc* Scheffe test, there were significant differences (*p* < 0.05) in the accuracy of emotion recognition between all emotions, except between fear (73% correct answers) and no emotion (74% correct answers).

The multinomial logistic regression was performed to ascertain how all the different elements of the stimulus (i.e., emotion, stimulus intensity, and stimulus gender) and the above-identified aspects of self-concept (i.e., intellectual/school status and general self-concept) could affect the correct emotion identifications. The logistic regression model was statistically significant [$\chi_{\left(8\right)}^{2}$ = 338.988, *p* = 0.000], explaining 71.4% of the variance in responses as a whole. All tested factors were found to contribute significantly to the correct identification of the emotions, as presented in Table 1.

**Table 1:** Components of the binomial logistic regression test and their contribution.

**Component**	**Wald**	**d.f**.	**Sig**.
Emotion	155.23	4	0.000
Intensity	108.32	2	0.000
Gender	11.833	1	0.001
Intellectual/school status	7.796	1	0.005
General self-concept	3.683	1	0.05

*This model explained 71.4% of the variance in correct detections of emotion*.

**Figure 4:**
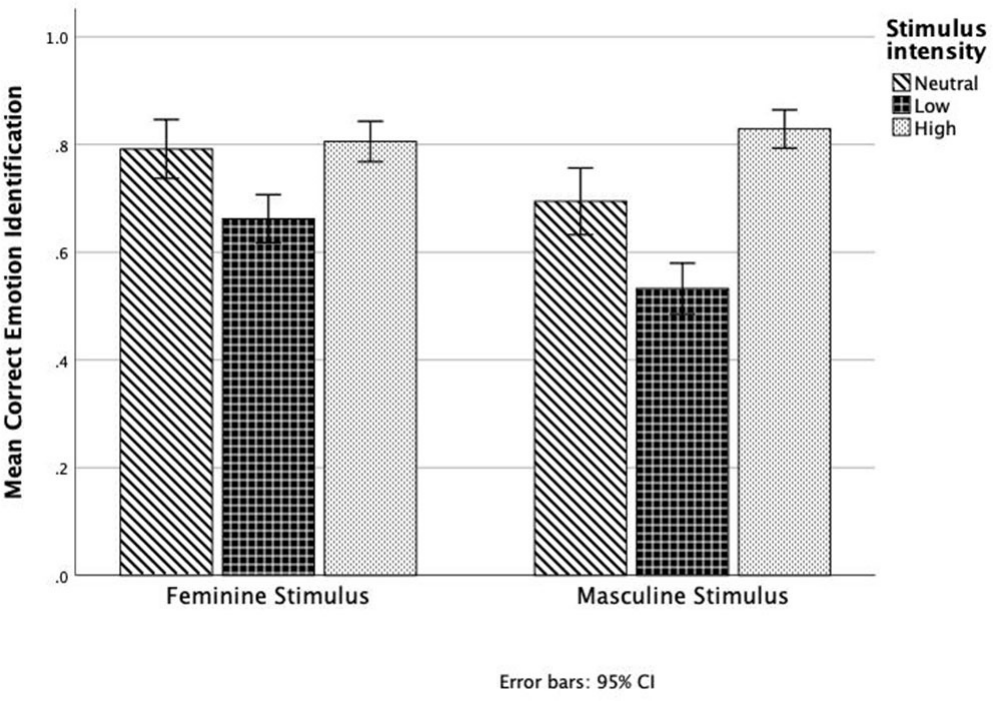
Proportion of correct emotion identification by stimulus gender and intensity.

Therefore, it can be concluded that several factors contribute to the correct detection of emotions from facial expressions in children. General self-concept and intellectual/school status self-concept contribute significantly. As can be seen in Figure 4, feminine stimuli seem to be more accurately identified than masculine stimuli. Lower intensity feminine stimuli had a score of 0.62, while lower intensity masculine stimuli had a score of only 0.51. Higher intensity stimuli had similar scores of 0.81 and 0.82 for feminine and masculine stimuli, respectively. Therefore, the differences in emotion identification by stimulus gender depended on stimulus intensity.

Separate binomial logistic regressions were performed *post-hoc*, with Bonferroni adjustments of *p* value, to ascertain the effects of general self-concept and intellectual/school status on the correct detections of emotion per emotion. A summary of significant effects is presented in Table 2.

**Table 2:** Significant effects of binomial logistic regression relating self-concept and emotion recognition.

**Test**	**Chi-Square**	**Sig**.	**% Variance**
General self-concept * Happiness	3.864	0.040	93.1
Intellectual/School status * Happiness	4.136	0.042	93.1
Intellectual/School status * Neutral	17.437	0.000	74.3

**Figure 5:**
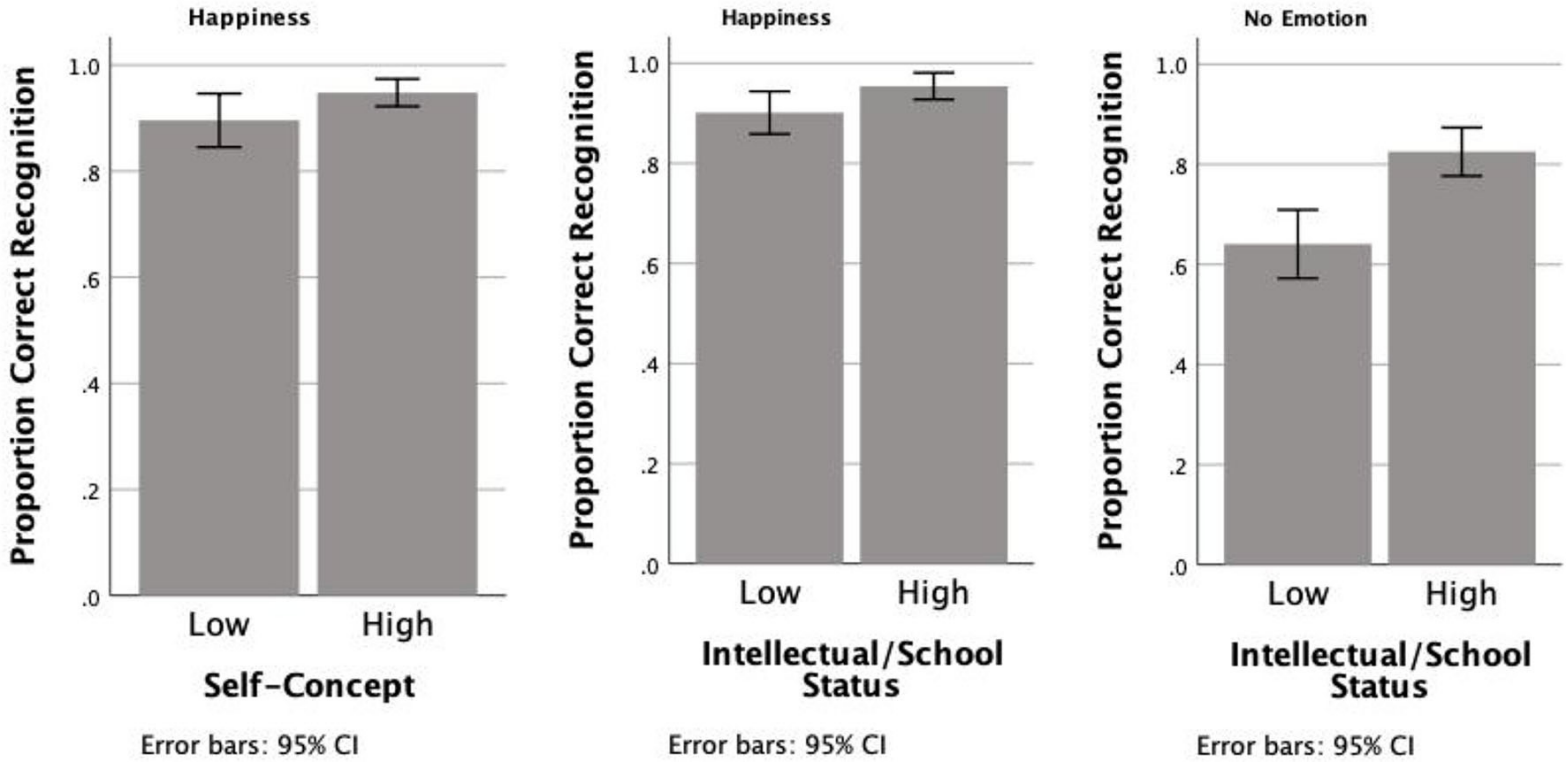
Proportion of correct emotion recognition by low- and high-self-concept children in the identification of happiness and no emotion.

It was found that the general self-concept affected how likely children were to correctly identify happiness (χ^2^ = 3.864, *p* = 0.04), and this model explained 93.1% of the variance in responses. From Figure 5, it can be seen that the higher self-concept levels were associated with greater rates of correct detection, although, in general, happiness was always well identified, and participants with lower self-concept had a score of 0.88, while participants with higher self-concept had a score of 0.96.

Similarly, the self-concept for intellectual/school status affected how correctly children identified happiness (χ^2^ = 4.136, *p* = 0.04), and this model also explained 93.1% of the variance in responses. From Figure 4, it can be seen that the children with the higher levels of self-concept for intellectual/school status had higher rates of correct responses (0.96) than children with lower levels of self-concept (0.88).

Interestingly, intellectual/school status also affected the correct detection of neutral faces (χ^2^ = 17.437, *p* = 0.000), and this model explained 74.3% of the variance in responses. In Figure 5, it is observed that the absence of emotion was not as correctly identified as happiness and that higher levels of self-concept for intellectual/school status were clearly associated with higher accuracy in this identification (0.83 for those with higher, as opposed to 0.63 for those with lower self-concept for intellectual status).

## Discussion

The aim of this study was to analyze how emotion recognition would be related to the self-concept of children. The results obtained revealed that there is a small but significant correlation between the overall recognition of emotions and one of the components of self-concept, which is the intellectual/school status. Furthermore, it was found that general self-concept is associated with the ability to correctly identify happy faces. Self-concept for intellectual/school status affected the recognition of happiness as well as neutral expressions. As secondary results, it was found that there are no statistically significant differences between the sexes regarding the ability to recognize emotions. It was also possible to verify that the ability to recognize emotions increases with the age of a child, in contrast to the self-concept, which decreased as the age increased. We also noted that not all emotions were equally identified. Happiness was the emotion most easily recognized by the children in our sample. In contrast, anger was the emotion which children were less able to recognize. Stimulus gender and intensity also affected the ability of participants to correctly identify emotions.

The first main surprising finding from this study was the association between the self-concept for intellectual/school status and the general ability to recognize emotions from facial expressions. This particular component of self-concept focuses on the mental aspects of self-concept. It includes not only items related directly to cognitive abilities (“I am a clever person,” “I am slow at finishing schoolwork,” “at school I am distracted thinking about other things,” “I forget what I learn,” “I am a good reader,” and “I am stupid about many things”) but also indicators of good quality adaptation to the school environment (“I am an important member of my classroom,” ”I can give a good impression within my class,” “My friends like my ideas,” and “My friends at school think I have good ideas”) and even items about good adaptation to the family (“I am an important member of my family”) and life expectations (“when I grow up I will be important”). This component of self-concept correlates highly with general scores of self-concept (Veiga, 2006). It would make sense that this component of self-concept would be more related to cognitive-emotional abilities than self-concept components related to anxiety, behavior, physical attributes, general happiness, or popularity.

Therefore, intellectual/school status is a component of self-concept that relates directly to how capable a child feels. This aspect may possibly be influenced by contributions from the family of a child and social experiences, as well as by their personal experiences of success during cognitive/school tasks. Regarding school and family experiences, it would be expected that the more the learning and cognitive efforts of a child are validated by an accepting environment the higher the self-concept for intellectual/school status. Therefore, it would make sense that this component would correlate with the ability to perceive emotions since it has been found that more accepted children do better at perceiving emotions (Vosk et al., 1983).

In another interpretation, it is possible that the child with a higher self-concept for intellectual/school status would actually have more developed cognitive abilities. One possible explaining factor would be that worse interactions at early ages could affect neuronal development, delaying the development of both cognitive and emotional skills. In this scenario, the correlation between this component of self-concept and the ability to recognize emotions could be mediated by elements or processes related to the intelligence of the child. This would somehow be in line with studies relating emotion perception abilities with cognitive abilities (Zaja and Rojahn, 2008) and emotional intelligence with school performance (Chamorro-Premuzic and Furnham, 2006; Ferrando et al., 2011). In fact, a recent review found that academic achievement in primary education was best predicted by academic self-concept, but emotional intelligence and other dimensions of self-concept also played a role (Herrera et al., 2020).

Another surprising finding of this study was how self-concept is associated with the ability to identify happiness. This finding might be related to the fact that this emotion was more correctly identified in general, which might indicate greater familiarity with it. Therefore, it might be argued that children with higher self-concept might be even more familiar with happy emotional expressions than other children, due to possibly having experienced more positive life experiences. It is also possible that children with better self-concept performed better at the task of differentiating between positive and negative emotional valence, which would particularly favor the recognition of happy faces. It is noteworthy that, throughout childhood, happiness is the first emotion to be well identified by children, followed by sadness and anger, and only at later stages of childhood anger (Adolphs, 2002b; Lawrence et al., 2015; Nelson and Mondloch, 2019). Therefore, the fact that children with higher general self-concept identified better happy faces might also mean that self-concept levels are associated with earlier development of emotion perception skills in general. Further studies, with older children, should inspect if, at later stages, the benefit in emotion recognition derived by high self-concept spreads to the recognition of other emotions.

In general, from our data, there seems to be an association between the aspects of self-concept and emotion recognition in children. This finding would support the simulation theory (Adolphs, 2002a; Shanton and Goldman, 2010). According to this theory, the perception of emotions in others would be mediated by the own internal representations of themselves of children. When observing facial expressions, the child might experience that same expression and use that internal experience to interpret its meaning in others.

As a secondary finding, it was observed that emotion recognition improves with the age of children. The older the children the better they are at discriminating the emotions presented. These results are in line with previous investigation (Gur et al., 2012; Nelson and Mondloch, 2019). Therefore, it is possible that these children in the future will fare better at discriminating emotions than they were at the moment. As stated above, a study with more participants should be devised to be able to analyze associations between self-concept and emotion recognition per age group during infancy.

It was additionally found that the values of self-concept decrease with increasing age regardless of gender. Thus, children, between 5 and 11 years old, may change the way they think about themselves, where life experiences may have a great influence, as well as changes in the environment that they may be subject to (Carneiro et al., 2003, cit in. Muniz and Fernandes, 2016). Previous study shows that, as age advances, the self-concept changes, as it does not remain static (Harter and Leahy, 2001), and may decrease or increase, despite our results showing a tendency to decrease.

There are practical implications to consider, from the results of this study. The main factor is that, so far, emotional well-being and self-concept have been seen as separate cognitive aspects, with less to no implications or effects in cognitive abilities and development of a child. In this study, the results we reported highlight that lower self-concepts might be associated with worse performance in some aspects of social cognition. From the practical standpoint, it becomes relevant to assess and promote the self-concept of children, both in family and in educational settings. The fact that self-concept for intellectual status stands out in its association with emotion recognition ability further highlights the need for the involvement of educators and teachers in particular. This would be, in any case, the most easily manipulable aspect of self-concept for that population, since the other aspects of self-concept relate to more stable internal factors such as anxiety, happiness and physical attributes, or social aspects, such as behavior and popularity.

In summary, it was found that self-concept might be associated with the ability to recognize emotions from facial expressions in children. In particular, there seems to be an association between intellectual/school status and emotion recognition. The emotion better recognized by children is happiness, and general self-concept affects particularly the correct identification of this emotion. The main limitation of this study is the relatively small number of participants and the lack of population diversity. However, the diversity did represent well the general population of the region where data were collected. It is also noteworthy that this sample was enough to obtain significant effects. A study with a more extended sample and additional metrics should follow to confirm the data reported in this study. Of interest, it would be the analysis of the quality of relationships with peers and family, as well as teaching methods of teachers and the cognitive abilities of children, which may act as mediators between self-concept and emotion recognition skills of children.

## Data Availability Statement

The raw data supporting the conclusions of this article will be made available by the authors, without undue reservation.

## Ethics Statement

The studies involving human participants were reviewed and approved by Ethics Committee of the University of the Azores. Written informed consent to participate in this study was provided by the participants' legal guardian/next of kin.

## Author Contributions

TC, JB, and CM planned this study. TC and JB collected the data, under the supervision of CM. CM analyzed the data, with support from TC and JB. TC and JB contributed equally to this project. All authors contributed to the article and approved the submitted version.

## Funding

This research was supported by the Center for Psychology at the University of Porto, Portuguese Science Foundation (FCT UIDB/00050/2020).

## Conflict of Interest

The authors declare that the research was conducted in the absence of any commercial or financial relationships that could be construed as a potential conflict of interest.

## Publisher's Note

All claims expressed in this article are solely those of the authors and do not necessarily represent those of their affiliated organizations, or those of the publisher, the editors and the reviewers. Any product that may be evaluated in this article, or claim that may be made by its manufacturer, is not guaranteed or endorsed by the publisher.
